# High-frequency power of heart rate variability can predict the outcome of thoracic surgical patients with acute respiratory distress syndrome on admission to the intensive care unit: a prospective, single-centric, case-controlled study

**DOI:** 10.1186/s12871-018-0497-5

**Published:** 2018-04-02

**Authors:** I-Chen Chen, Chew-Teng Kor, Ching-Hsiung Lin, Jane Kuo, Jang-Zern Tsai, Wen-Je Ko, Cheng-Deng Kuo

**Affiliations:** 1 Intensive Care Units, National Taiwan University Hospital, Taipei, Taiwan; 2 Internal Medicine Research Center, Changhua Christian Hospital, Changhua, Taiwan; 3 Division of Chest Medicine, Department of Internal Medicine, Changhua Christian Hospital, Changhua, Taiwan; 4 Department of Respiratory Care, College of Health Sciences, Chang Jung Christian University, Tainan, Taiwan; 5 School of Dentistry, College of Dental Medicine, Kaohsiung Medical University, Kaohsiung, Taiwan; 6 Department of Electrical Engineering, National Central University, Jung-Li Taoyuan, Taiwan; 7 Laboratory of Biophysics, Department of Medical Research, Taipei Veterans General Hospital, Taipei, Taiwan

**Keywords:** Acute respiratory distress syndrome, Heart rate variability, Outcome, Mortality

## Abstract

**Background:**

The morbidity and mortality of acute respiratory distress syndrome (ARDS) remains high, and the strategic focus of ARDS research has shifted toward identifying patients at high risk of mortality early in the course of illness. This study intended to identify the heart rate variability (HRV) measure that can predict the outcome of patients with ARDS on admission to the surgical intensive care unit (SICU).

**Methods:**

Patients who had lung or esophageal cancer surgery were included either in the ARDS group (*n* = 21) if they developed ARDS after surgery or in the control group (*n* = 11) if they did not. The ARDS patients were further stratified into survivors and non-survivors subgroups according to their outcomes. HRV measures of the patients were used for statistical analysis.

**Results:**

The mean RR interval (mRRI), high-frequency power (HFP) and product of low-/high-frequency power ratio tidal volume and tidal volume (LHR*V_T_) were significantly lower (*p* < 0.05), while the normalized HFP to V_T_ ratio (nHFP/V_T_) was significantly higher in the ARDS patients (*p* = 0.011). The total power (TP), low-frequency power (LFP), HFP and HFP/V_T_ were all significantly higher in the non-survived ARDS patients, whereas Richmond Agitation-Sedation Scale (RASS) was significantly lower in the non-survived ARDS patients. After adjustment for RASS, age and gender, firth logistic regression analysis identified the HFP, TP as the significant independent predictors of mortality for ARDS patients.

**Conclusions:**

The vagal modulation of thoracic surgical patients with ARDS was enhanced as compared to that of non-ARDS patients, and the non-survived ARDS patients had higher vagal activity than those of survived ARDS patients. The vagal modulation-related parameters such as TP and HFP were independent predictors of mortality in patients with ARDS on admission to the SICU, and the HFP was found to be the best predictor of mortality for those ARDS patients. Increased vagal modulation might be an indicator for poor prognosis in critically ill patients following thoracic surgery.

## Background

Acute respiratory distress syndrome (ARDS) is a life threatening lung condition that affects both medical and surgical patients [1, 2]. Postoperative ARDS is a recognized complication after lung resection or esophagectomy for cancer [3–5]. The incidence of ARDS after these procedures was 1–3% [3, 6], and the overall hospital mortality rates associated with ARDS were over 40% [5–8].

Many scoring systems have been developed and used in the intensive care unit (ICU) to monitor the treatment and predict the outcomes of patients with ARDS. For instance, Navarrete-Navarro et al. [9] showed that the ICU mortality of trauma patients with ARDS was related to the Acute Physiology and Chronic Health Evaluation (APACHE) III score and PaO_2_/FIO_2_ on the 3rd day. Luecke et al. [10] showed that the APACHE II was the only clinical predictor for mortality on admission to the ICU. Bauman et al. [11] showed that the lung injury score was reliable for predicting mortality in critically ill surgical patients.

The autonomic nervous system is important for the maintenance of homeostasis. A wide range of pathophysiologic conditions can influence the balance in the autonomic nervous system. Heart rate variability (HRV) refers to the variation of interbeat intervals due to respiration and other physiological inputs. The assessment of HRV is based on the analysis of consecutive normal R-R intervals, and may provide clinicians with quantitative information about cardiac autonomic nervous modulation. HRV can be quantified by time domain and frequency domain parameters. Thus, HRV analysis has become a useful non-invasive technique for evaluation of cardiac autonomic nervous modulation in many kinds of illnesses [12].

Previous studies have reported that HRV measures are decreased in survivors of cardiac arrest [13]. Some studies found that the standard deviation of normal-to-normal QRS intervals (SDNN), one of six commonly used time-domain HRV measures, is a significant parameter for long-term prognosis in patients with acute myocardial infarction [14, 15]. Tibby et al. [16] showed that the loss of HRV occurs with increasing organ failure, and that this effect is better demonstrated by the power-law model of HRV than by other measures of HRV. Annane et al. [17] reported that the low-frequency (LF) component of the oscillations in heart rate and diastolic blood pressure variability are dramatically reduced in patients with septic shock, and that the decrease in the LF component may identify septic patients with a high level of sympathetic activation. Pontet et al. [18] demonstrated that reduction of HRV in patients on ICU admission may be useful in identifying septic patients at risk for the development of multiple organ dysfunction syndromes. In patients with severe sepsis, depressed sympathetic modulation has been found to be indicative of poor outcome [19]. In patients in the emergency department who have been successfully resuscitated after out-of-hospital cardiac arrest, the initial HRV measures resemble those of severe sepsis and are capable of predicting 24-h mortality [20, 21]. In the emergency department, the characteristics of HRV can predict impending septic shock in patients with sepsis [22]. These studies suggest that HRV measures might be useful for the monitoring of patients with various kinds of diseases, including ARDS, and for predicting their prognosis.

Despite significant advances in the management of patients with ARDS in the past fifty years, the morbidity and mortality of ARDS remains high, and the strategic focus of ARDS research has shifted toward identifying patients with or at high risk of ARDS early in the course of their illnesses [23]. Thus, the aim of this study was to evaluate the possibility of using HRV measures to predict the outcome in patients with ARDS on admission to the surgical intensive unit (SICU).

## Methods

### Research Design

This was a prospective, single-centric, case-controlled study. The study protocol has been approved by the Institutional Review Boards of National Taiwan University Hospital (NTUH200808065R) and Taipei Veterans General Hospital (VGHIRB97–01-02A), and written informed consent was obtained from the patients or the next of kin of the patients before their enrollment in the study.

### Study Participants

This study was conducted in the SICU of National Taiwan University Hospital. All the recruited patients were over 18 years old. They were partitioned into an ARDS group and a control group. The ARDS group consisted of patients who had received thoracic surgery because of lung or esophageal cancer and were transferred to the SICU for intensive care because of ARDS later. The control group consisted of patients who had received thoracic surgery because of lung or esophageal cancer and were transferred to the SICU for post-operative care. The ARDS was diagnosed according to the Berlin Definition [2]. A draft definition proposed 3 mutually exclusive categories of ARDS based on the degree of hypoxemia: mild (200 mmHg < PaO_2_/FIO_2_ ≤ 300 mmHg), moderate (100 mmHg < PaO_2_/FIO_2_ ≤ 200 mmHg), and severe (PaO_2_/FIO_2_ ≤ 100 mmHg) with 4 ancillary variables for severe ARDS: radiographic severity, respiratory system compliance (≤ 40 mL/cm H_2_O), positive end-expiratory pressure (≥ 10 cm H_2_O), and corrected expired volume per minute (≥ 10 L/min). Patients with severe coronary artery disease, persistent arrhythmia, cardiac pacing, diabetes mellitus, cerebral vascular accident, or major diseases of kidney or autoimmune system were excluded from the study.

### Physiological Measurements

All patients required intubation and mechanical ventilation. The acute lung injury score (ALIS) [24], alveolar-arterial oxygen difference (AaDO_2_), APACHE II [25], demographic data, vital signs, medications, ventilator parameters including respiratory rate (RR), inspiratory pressure (P_insp_), inspiration time (T_insp_), tidal volume (V_T_), positive end-expiratory pressure (PEEP), minute ventilation (MV), fraction of inspired oxygen (FIO_2_), dynamic compliance (Cdyn), and arterial blood gases data were recorded within 4 h of admission to the SICU. Electrocardiographic (ECG) signals were recorded for 12 min in supine position using the MP35 multichannel recorder (BIOPAC Systems, Goleta, CA, USA). The primary outcome measure was survival at discharge from the SICU. Patients who survived the ARDS were classified as the survivors, while those patients who did not survive the ARDS were classified as non-survivors. Clinical information obtained in the SICU and the outcomes of the patients were blinded to the investigator who performed the HRV analysis and clinical data collection.

### HRV Analysis

Power spectral density (PSD) analysis by using parametric autoregressive modeling or non-parametric Fourier transform provides the basic information about the power (variance) distributes of experimental data as a function of frequency. The Fourier transform decomposes a signal into its constituent frequencies. The Fourier transform of a signal is a complex-valued function of frequency, whose absolute value represents the amount of that particular frequency present in the signal. The power of a particular frequency band is calculated by integrating the PSD within that frequency band. Thus, the Fourier transform is the frequency domain representation of the original signal [26].

The method used for the HRV analysis adhered to the standards laid down by the Task Force of the European Society of Cardiology and the North American Society of Pacing and Electrophysiology [12]. All ectopic beats were removed and the missing data were replaced by interpolated beats derived from the nearest valid data. Any patient with more than 5% of ectopic beats was excluded from the study. The last 512 stationary RR intervals (RRI) were used for HRV analysis.

The time domain HRV measures included mRRI, standard deviation of RRI (SD_RR_), coefficient of variation of RRI (CV_RR_), and root mean squared successive difference of RRI (RMSSD). The power spectrum of RRI was obtained by means of fast Fourier transformation (Mathcad 15, Mathsoft Inc.). The areas under the spectral peaks within the range of 0.01–0.04 Hz, 0.04–0.15 Hz, 0.15–0.5 Hz, and 0.01–0.5 Hz were defined as the very low-frequency power (VLFP), LFP, high frequency power (HFP), and TP, respectively. The normalized VLFP (nVLFP = VLFP/TP × 100) was used as the index of renin-angiotensin-aldosterone modulation, thermal regulation and vagal withdrawal; the normalized LFP (nLFP = LFP/TP × 100) as the index of combined sympathetic and vagal activities; the normalized HFP (nHFP = HFP/TP × 100) and HFP as the index of cardiac vagal activity; and the low-/high-frequency power ratio (LHR = LFP/HFP) as the index of sympathovagal balance. Since the HFP and nHFP can be influenced by carbon dioxide, respiratory rate and tidal volume (V_T_) [27], the HFP/V_T_ and nHFP/V_T_ were used as the V_T_-corrected indices of vagal modulation, and the LHR*V_T_ was used as the V_T_-corrected index of sympathovagal balance [20].

### Statistical Analysis

Continuous variables were compared between the two groups of patients using Mann–Whitney U test. Fisher’s exact test was used for comparisons of categorical data. Pearson product moment analysis was performed to find the correlations among HRV measures and clinical variables of the patients. The Firth logistic regression and multiple linear regression models were employed to identify the most important independent predictors of mortality and ICU stay day, respectively. Firth’s penalized likelihood approach was used to reduce the bias of the parameter estimates due to small sample size. Significant HRV measures in the univariate analyses of differences between the two subgroups of ARDS patients were included in the multivariate analyses. In this study, the 10 events per variable (EPV − 10) rule [28, 29] was used in logistic regression model to avoid the overfitting problem, which is a generally accepted criterion in multivariable analysis. With a sample size of 32 patients (11 patients in the control group and 21 patients in the ARDS group), the inclusion of only three variables in the multivariate analysis to ensure adequate statistical power is reasonable. The binary receiver operating characteristic (ROC) curves for statistically independent variables associated with mortality were drawn. All statistical analyses were performed using R software (version i386 3.3.2, https://www.r-project.org/) and the contributed R package logistic for Firth’s penalized-likelihood logistic regression. A *p* < 0.05 was considered statistically significant.

## Results

During the 2 years study period, 22 patients developed ARDS among the 817 patients that received lung or esophageal surgery. Thus the incidence rate of ARDS after thoracic surgery for lung or esophageal surgery was 2.69%. Among those 22 ARDS patients, 15 were included in the ARDS group while 7 were not because of arrhythmia. Six patients who were not operated during this admission but had thoracic surgery within past 1 year and developed ARDS due to rapidly progressing pneumonia were also admitted and included in the ARDS group. Thus, there were 11 patients in the non-ARDS group and 21 patients in the ARDS group.

Fentanyl (5~ 10 μg·hr^-1^) was administered to the patients during the post-operative period for post-surgical pain killing. The Fentanyl was given to the non-ARDS patients for 24–48 h, while it was given to the ARDS patients for more than 48 h. Midazolam (1~ 2 mg/h) was used to maintain Richmond Agitation-Sedation Scale (RASS) 0 ~ − 2 in both groups, and dopamine and norepinephrine (0.05 ~ 0.11 μg·hr^− 1^·min^− 1^) were used in 8 patients in the ARDS group to keep mean blood pressure > 60 mmHg to maintain proper cerebral perfusion pressure. The ECG recordings were taken within 4 h of transfer to the SICU in all patients.

In the control group, 6 patients had lung cancer and 5 patients had esophageal cancer. In the ARDS group, 9 patients had lung cancer, 5 patients had esophageal cancer, 1 patient had esophageal rupture, and 6 patients had pneumonia-induced ARDS after the thoracic surgery. Six patients out of 11 patients in the control group were classified as American Society of Anesthesiologists (ASA) I, and 5 patients as ASA II. For the 21 patients in the ARDS group, 1 patient was classified as ASA I, 5 patients as ASA II, 4 patients as ASA III. Six patients in the ARDS group had no ASA classification because they were not operated during this admission, but had thoracic surgery within past 1 year, and were admitted due to ARDS caused by rapidly progressing pneumonia (Table 1).

**Table 1:** Comparison between patients with and without ARDS

	Non-ARDS	ARDS	*p* value
	(*n* = 11)	(*n* = 21)	
Clinical data			
Age (year)	62 (57–67)	63 (50–71)	0.889
Body height (cm)	154 (151–171)	167 (163–170)	0.176
Body weight (kg)	65 (57–76)	63 (58–72)	0.953
APACHE II	9 (6–12)	16 (11–19)	0.002
Pre-operation			
Lung cancer/Esophageal cancer	6/5	9/5	0.697
Hypertension (Yes/No)	3/8	4/17	0.667
ASA	1 (1–2)	2 (2–3)	0.005
VATS/open surgery	11/0	12/3	0.667
Blood transfusion (Yes/No)	5/6	5/10	0.457
Airway management	9/2	10/5	0.540
(double-lumen tube/bronchus blocker)			
Anesthesia (volatile/intravenous)	11/0	15/0	1.000
Surgery time (minute)	255 (245–450)	250 (165–375)	0.474
Intraoperative I/O (ml)	2000 (1500–2400)	1300 (650–1800)	0.015
ICU stay days (day)	4 (2–4)	25 (13–42)	< 0.001
RASS	0 (0–0)	-1 (− 1–0)	0.001
Ventilator free hours	0 (0–0)	48 (24–68)	NA
Medications			
Midazolam (Yes/No)	4/7	20/1	< 0.001
Fentanyl (Yes/No)	11/0	21/0	1.000
Norepinephrine (Yes/No)	0/11	8/13	0.021
Dopamine (Yes/No)	0/11	3/17	0.531
Ventilator settings			
FIO_2_	0.40 (0.40–0.40)	0.70 (0.60–0.93)	< 0.001
PEEP (cmH_2_O)	5.0 (5.0–7.6)	10.0 (9.7–12.0)	< 0.001
RR (bpm)	12 (12–13)	17 (16–27)	< 0.001
P_insp_ (cmH_2_O)	20 (20–20)	20 (17–22)	1.000
T_insp_ (sec)	0.9 (0.9–1.0)	0.9 (0.8–1.0)	0.546
V_T_ (ml)	490 (470–617)	420 (380–540)	0.004
MV (l/min)	6.0 (5.6–8.3)	9.0 (6.6–10.6)	0.142
Cdyn (ml/cmH_2_O)	25.0 (23.5–32.0)	21.1 (18.0–26.7)	0.010
Arterial gas analysis			
pH	7.42 (7.37–7.46)	7.44 (7.39–7.47)	0.524
PaO_2_ (mmHg)	171 (133–230)	79 (67–97)	< 0.001
PaCO_2_ (mmHg)	32 (27–39)	39 (32–42)	0.043
HCO_3_^−^ (mmol/l)	20 (19–22)	26 (21–28)	0.017
PaO_2_/FIO_2_ (mmHg)	477 (343–559)	106 (86–171)	< 0.001
AaDO_2_ (mmHg)	47 (34–104)	368 (266–531)	< 0.001
Time domain HRV measures			
mRRI (ms)	684 (589–874)	604 (524–649)	0.017
SD_RR_ (ms)	32 (28–43)	29 (25–36)	0.132
CV_RR_ (%)	4.7 (4.5–4.9)	4.5 (4.5–5.0)	0.284
RMSSD (ms)	31 (28–41)	29 (25–37)	0.204
Frequency domain HRV measures			
TP (ms^2^)	267 (206–614)	212 (134–316)	0.068
VLFP (ms^2^)	29 (22–92)	21 (11–47)	0.068
LFP (ms^2^)	73 (48–168)	54 (39–78)	0.052
HFP (ms^2^)	171 (131–387)	122 (83–176)	0.043
HFP/V_T_ (ms^2^/ml)	0.35 (0.22–0.81)	0.31 (0.21–0.44)	0.383
nVLFP (nu)	11.0 (8.6–14.2)	9.3 (7.9–14.1)	0.341
nLFP (nu)	27.4 (24.5–27.8)	27.9 (26.9–28.5)	0.361
nHFP (nu)	62.0 (60.0–64.0)	62.9 (59.4–63.6)	0.890
nHFP/V_T_ (nu/ml)	0.11 (0.10–0.13)	0.15 (0.12–0.18)	0.011
LHR	0.45 (0.43–0.47)	0.45 (0.44–0.48)	0.937
LHR*V_T_ (1/ml)	228 (209–282)	186 (149–238)	0.010

Data are presented as medians (25% - 75% interquartile range). *APACHE II*: Acute Physiology and Chronic Health Evaluation II; *ASA*: American Society of Anesthesiologists; *VATS*: Video-Assisted Thoracic Surgery; *I/O*: intake/output; *RASS*: Richmond Agitation-Sedation Scale; *FIO2*: fraction of inspired oxygen; *PEEP*: positive end-expiratory pressure; *RR*: respiratory rate; *Pinsp*: inspiratory pressure; *Tinsp*: inspiration time; *VT*: tidal volume; *MV*: minute ventilation; *Cdyn*: dynamic compliance; *PaO2*: partial pressure of arterial oxygen; *PaCO2*: partial pressure of arterial carbon dioxide; *HCO3*: bicarbonate; *AaDO2*: alveolo-arterial oxygen difference; *ALI score*: acute lung injury score; *RASS*: Richmond Agitation-Sedation Scale; *mRRI*: mean RR interval; *SDRR*: standard deviation of RR intervals; *CVRR*: coefficient of variation of RR intervals; *RMSSD*: root mean squared successive difference; *TP*: total power; *VLFP*: very low-frequency power; *LFP*: low-frequency power; *HFP*: high-frequency power; *nVLFP*: normalized VLFP; *nLFP*: normalized LFP; *nHFP*: normalized HFP; body weight; *LHR*: 4 low-/high- frequency power ratio; *ml*: milliliter; *bpm*: beats per minute; *ms*: milliseconds; *nu*: normalized unit; *NA*: not assessed. The "*" in "LHR*V_T_" denotes multiplication

Ten patients in the control group received video-assisted thoracic surgery (VATS) for lobectomy or esophageal resection, and 1 patient received VATS for laminectomy because of lung cancer metastasis. In the ARDS group, 12 patients received VATS for lobectomy or esophageal resection, 3 patients received open lung surgery, and 6 patients received thoracic surgery within the past 1 year who were admitted to the SICU because of ARDS caused by pneumonia (Table 1).

In the control group, 2 patients received bronchus blocker, and 9 patients received double-lumen intubation. In the ARDS group, 15 patients received thoracic surgery during this admission; among them, 5 patients received bronchus blocker and 10 patients received double-lumen intubation. All the 26 patients who received thoracic surgery during this admission were operated under Desflurane and Sevoflurane anesthesia (minimum alveolar concentration: 0.8–1.1 Vol%). Five out of the 11 patients in the control group received blood transfusion; the transfusion rate in the control group was 45.5%. In the ARDS group, 6 out of the 15 patients who received thoracic surgery during this admission had blood transfusion. Therefore, the transfusion rate was 40% in the ARDS group. The duration of surgery in the control group was 255 (245–450) minutes (median and interquartile range), whereas the duration of surgery in the ARDS group was 250 (165–375) minutes (Table 1).

Tables 1 shows that the ARDS patients had higher RR, required higher FIO_2_ and PEEP during ventilation. The V_T_, Cdyn, PaO_2_ and PaO_2_/FIO_2_, intraoperative intake/output (I/O) of the ARDS patients was significantly lower (*p* < 0.01), whereas the arterial partial pressure of carbon dioxide (PaCO_2_), bicarbonate (HCO_3_^−^), ASA classification, and AaDO_2_ of the ARDS patients were significantly higher than those of non-ARDS patients (*p* < 0.05). The mRRI, HFP and LHR*V_T_ were significantly lower (*p* < 0.05), while the nHFP/V_T_ was significantly higher in the ARDS patients (*p* = 0.011). Table 2 shows that there were no significant differences in most ventilator settings, disease severity scores including ALIS, AaDO_2_, APACHE II, intraoperative anesthesia control, blood transfusion rate, fluid balance, and blood gas data between the survivors and non-survivors of ARDS. However, the P_insp_, PaCO_2_, HCO_3_^−^, TP, LFP, HFP, and HFP/V_T_ of the non-survivors were higher than those of the survivors (p < 0.05), and RASS of the non-survivors was significantly lower than that of the survivors (*p* = 0.040).

**Table 2:** Comparison between survivors and non-survivors in patients with ARDS

	Survivors	Non-survivors	*p* value
	(*n* = 16)	(*n* = 5)	
Clinical data			
Age (year)	59.5 (41.8–70.0)	71.0 (59.5–77.5)	0.136
Body height (cm)	167 (163–170)	166 (154–170)	0.835
Body weight (kg)	64 (59–71)	62 (57–80)	0.773
ALI score	13 (10–14)	13 (12–15)	0.557
APACHE II	14 (10–19)	18 (14–29)	0.185
Pre-operation			
Hypertension (Yes/No)	2/14	2/3	0.228
ASA	2 (2–3)	2 (2–3)	0.768
VATS/open surgery	8/2	4/1	0.660
Surgery time (minute)	210 (280–457)	320 (220–345)	1.000
Blood transfusion (Yes/No)	5/5	1/0	0.719
Airway management	7/3	3/2	0.768
(double-lumen tube/bronchus blocker)			
Anesthesia (volatile/intravenous)	10/0	5/0	1.000
Intraoperative I/O	780 (1350–1800)	1300 (600–1600)	0.679
ICU stay days	21 (11–44)	25 (20–43)	0.535
RASS	-1 (−1–0)	−2 (−2 - -1)	0.040
Ventilator free hours	48 (24–68)	0 (0–10)	0.177
Re-thoracotomy (Yes/No)	0/16	0/5	1.000
Re-intubation rate (Yes/No)	5/16	2/0	0.780
SOFA score	8 (7–10)	7 (7–8)	0.313
WBC	13.3 (7.7–13.4)	12.9 (11.1–14.0)	0.905
CRP	21.5 (10.9–22.6)	12.4 (11–19.7)	0.495
Medications			
Midazolam (Yes/No)	15/1	5/0	1.000
Fentanyl (Yes/No)	16/0	5/0	1.000
Norepinephrine (Yes/No)	6/10	2/3	1.000
Dopamine (Yes/No)	2/13	1/4	0.842
Ventilator settings			
FIO_2_	0.70 (0.56–0.94)	0.65 (0.60–0.93)	0.901
PEEP (cmH_2_O)	10.0 (8.5–12.0)	10.2 (9.7–13.5)	0.552
RR (bpm)	16.5 (15.3–23.0)	26.0 (20.0–27.5)	0.156
P_insp_ (cmH_2_O)	18.0 (15.3–20.0)	22.0 (20.0–22.5)	0.039
T_insp_ (sec)	0.9 (0.8–1.0)	0.9 (0.8–1.0)	0.548
V_T_ (ml)	425 (385–545)	390 (305–493)	0.385
MV (l/min)	8.0 (6.6–10.5)	10.2 (6.9–12.9)	0.363
Cdyn (ml/cmH_2_O)	21.6 (18.5–32.0)	19.0 (13.9–23.4)	0.231
Arterial gas analysis			
pH	7.42 (7.38–7.45)	7.47 (7.42–7.49)	0.074
PaO_2_ (mmHg)	78 (67–97)	90 (66–103)	0.772
PaCO_2_ (mmHg)	38 (29–41)	40 (39–51)	0.043
HCO3^−^ (mmol/l)	24 (19–27)	30 (28–33)	0.003
PaO_2_/FIO_2_ (mmHg)	115 (84–180)	106 (83–171)	0.901
AaDO_2_ (mmHg)	384 (256–536)	352 (275–516)	0.967
Time domain HRV measures			
mRRI (ms)	569 (496–636)	655 (600–761)	0.063
SD_RR_ (ms)	28 (23–34)	31 (30–41)	0.063
CV_RR_ (%)	4.51 (4.47–4.77)	4.98 (4.49–6.03)	0.409
RMSSD (ms)	27 (23–35)	34 (31–44)	0.052
Frequency domain HRV measures			
TP (ms^2^)	161 (113–246)	274 (234–529)	0.023
VLFP (ms^2^)	13 (9–55)	22 (20–70)	0.231
LFP (ms^2^)	47 (32–59)	75 (64–127)	0.023
HFP (ms^2^)	101 (71–133)	175 (148–335)	0.012
HFP/V_T_ (ms^2^/ml)	0.23 (0.18–0.32)	0.64 (0.35–0.82)	0.007
nVLFP (nu)	9.7 (7.9–14.3)	8.9 (6.3–14.4)	0.710
nLFP (nu)	27.9 (26.9–28.8)	27.4 (22.4–28.3)	0.509
nHFP (nu)	62.0 (59.4–63.5)	63.7 (57.5–71.0)	0.127
nHFP/V_T_ (nu/ml)	0.15 (0.10–0.16)	0.17 (0.12–0.24)	0.386
LHR	0.45 (0.44–0.48)	0.43 (0.33–0.50)	0.127
LHR*V_T_ (1/ml)	189 (179–254)	161 (95–238)	0.302

Data are presented as medians (25% - 75% interquartile). *ALI*: acute lung injury score; *RASS*: Richmond Agitation-Sedation Scale; *RASS*: Richmond Agitation-Sedation Scale; *SOFA*: Sequential Organ Failure Assessment; *WBC*: white blood cells; *CRP*: C-reactive protein. Other abbreviations are the same as those depicted in Table 1. The "*" in "LHR*V_T_" denotes multiplication

Pearson product moment analysis among the significantly different HRV measures between the survived and non-survived ARDS patients showed that the TP, LFP, HFP, and HFP/V_T_ also correlated significantly and positively with one another (Table 3), but these HRV measures did not correlate with RASS.

**Table 3:** Correlation coefficient in the Pearson product moment correlation analyses of HRV parameters and RASS in patients with ARDS

	LFP	HFP	HFP/V_T_	RASS
TP	0.958*	0.955*	0.793*	0.181
LFP		0.870*	0.725*	0.235
HFP			0.878*	0.019

*p < 0.05. *HFP*: high-frequency power; *TP*: total power; *LFP*: low-frequency power; *V*_*T*_: tidal volume; *RASS*: Richmond Agitation-Sedation Scale

Firth logistic regression analysis was used to assess the risk of mortality (dependent variable) in ARDS patients. The ASA classification, intraoperative anesthesia, fluid balance, drugs, etc., were not significantly different between the survivors and non- survivors of ARDS; only the RASS of the non-survivors was significantly lower than that of the survivors (Table 2). In univariate analysis, the TP, HFP and RASS were found to be the significant predictors of mortality for ARDS patients in the SICU (*p* < 0.05) (Table 4). Since the TP and HFP were highly correlated with each other while the RASS did not correlate with either TP or HFP (Table 3), two separate models in multivariate analysis including HFP and TP adjusted for RASS, age and gender were used. After adjustment for RASS, age and gender, the HFP (*p* = 0.025) and TP (*p* = 0.024) were found to be the significant independent predictors of mortality in ARDS patients in the SICU (Table 4).

**Table 4:** Firth logistic regression analyses for predicting the mortality of patients with ARDS

Univariate Model			Multivariate model 1 Adjusted for HFP, RASS, age and gender		Multivariate model 2 Adjusted for TP, RASS, age and gender	
OR (95% CI)		p value	OR (95% CI)	*p* value	OR (95% CI)	p value
HFP	1.013 (1.002,1.03)	0.020	1.016 (1.002,1.149)	0.025		
TP	1.007 (1.00,1.02)	0.047			1.010 (1.001,1.065)	0.024
LFP	1.021 (0.998,1.05)	0.080				

*OR*: odds ratio; 95% *CI*: 95% profile-likelihood confidence intervals; *HFP*: high-frequency power; *TP*: total power; *LFP*: low-frequency power

**Fig. 1:**
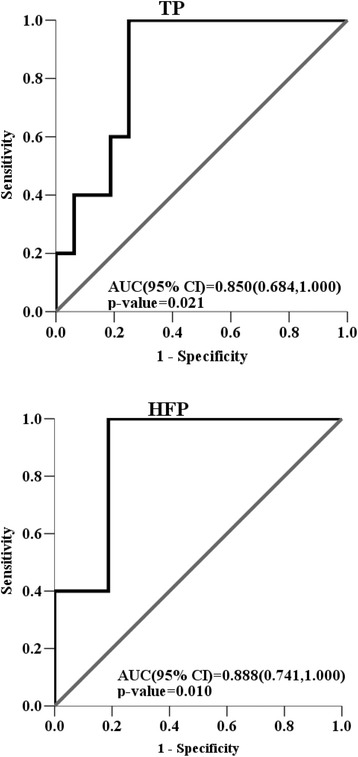
The areas under the ROC curves (AUC) and 95% confidence intervals (CI) of TP and HFP in predicting the mortality of ARDS patients on admission to the SICU were 0.850 (95% CI = 0.685–1.000; *p* = 0.021), and 0.888 (95% CI = 0.741–1.000; *p* = 0.010), respectively

As depicted in Fig. 1, the AUCs and 95% confidence intervals (CI) of TP and HFP in predicting mortality in ARDS patients on admission to the SICU were 0.850 (95% CI = 0.685–1.000; *p* = 0.021) and 0.888 (95% CI = 0.741–1.000; *p* = 0.010), respectively. Among all variables assessed in this study, HFP was the best predictor in predicting mortality in ARDS patients on admission to the SICU.

Linear regression analysis was used to assess the association between ICU stay days (dependent variable) and HRV measures in ARDS patients. It was found that the TP (*p* = 0.681), LFP (*p* = 0.740), HFP (*p* = 0.686) and RASS (*p* = 0.108) were not significantly associated with the days of ICU stay of ARDS patients in the SICU (Table 5).

**Table 5:** Linear regression analyses for predicting the ICU stay days in patients with ARDS

	Univariate analysis		
	Unstandardized coefficients		*p* value
	B	Standard Error	
TP	0.005	0.011	0.681
LFP	−0.008	0.024	0.740
HFP	−0.005	0.012	0.686
RASS	−0.615	0.361	0.108

The ICU stay days were analyzed in ln-transformation manner due to non-normality property. *HFP*: high-frequency power; *TP*: total power; *LFP*: low-frequency power

## Discussions

Spectral HRV analysis allows us to differentiate between branches of the autonomic nervous system [12] and to assess the autonomic modulation of critically ill patients [16–22]. This study compared the clinical characteristics and HRV measures between non-ARDS and ARDS patients, and between ARDS patients who survived and did not survive. It was found that the HFP was significantly lower (*p* = 0.043) while the nHFP/V_T_ was significantly higher (*p* = 0.011) in the ARDS group compared with those in the non-ARDS group, and that the non-survived ARDS patients had higher TP, LFP, HFP, and HFP/V_T_ than the survived ARDS patients had. After adjustment for RASS, age and gender, firth logistic regression analysis identified HFP and TP as the independent predictors of mortality in ARDS patients on admission to the SICU. The HFP was found to be the best predictor of mortality in ARDS patients.

Many studies have proposed that clinical laboratory data and HRV measures can be used to predict the prognosis of certain diseases. For instance, Luhr et al. [30] showed that age and acute physiologic score were associated with mortality in the ARDS patients. Phillips et al. [31] had shown that the lung injury score of non-survivors was significantly higher than that of survivors in patients with ARDS. Swaroopa et al. [32] had shown that the APACHE II score was significantly higher in non-survivors as compared to survivors in patients with ARDS. In this study it was found that the APACHE II, ALIS, PaO_2_/FIO_2_ and AaDO_2_ were not significantly different between the survived and non-survived ARDS patients; instead, the HFP and TP were found to be the independent predictors of mortality in ARDS patients on admission to the SICU (*p* < 0.05). This result suggested that the non-invasive HRV indices such as HFP and TP were more sensitive than the conventional scores or indices such as APACHE II, ALIS, PaO_2_/FIO_2_ and AaDO_2_ in predicting the outcome of surgical patients with ARDS. Because of its non-invasiveness and close relation with the autonomic control of heart rate, HRV is often used in the monitoring and prognosis of critically ill patients. For instance, Longin et al. [33] studied the differences in HRV variables during extracorporeal membrane oxygenation (ECMO) therapy between survivors (*n* = 6) and non-survivors (*n* = 7) and found that the survivors showed a tendentious, but not significant, decrease in HRV variables (ECMO-start vs. ECMO-stop), while the non-survivors had elevated HRV values. Winchell et al. [34] showed that both low TP and LHR were associated with increased mortality, while sympathetic predominance favored survival in the ICU setting. Chen et al. [35] found that a worse oxygenation status is associated with increased cardiac vagal and decreased cardiac sympathetic activities in patients with chronic obstructive pulmonary disease (COPD). For patients with sepsis in the emergency department, Chen et al. [36] also found that the SDNN, TP, VLFP, LFP, and LHR were all significantly lower while the nHFP was significantly higher in the non-survivors of sepsis. In another study, Chen et al. [20] further showed that the LFP, nLFP and LHR were significantly lower while the RMSSD, HFP, HFP/V_T_, nHFP, and nHFP/V_T_ were significantly higher in the non-survivors of patients with out-of-hospital cardiac arrest. These results suggested that HRV measures can be used in the monitoring and prognosis of critically ill patients, and that increased cardiac vagal and decreased cardiac sympathetic activities might be indicative of poor prognosis. In accordance with these studies, the present study also found that the ARDS patients had higher vagal modulation and smaller sympathetic modulation than the non-ARDS patients, and that the non-survived ARDS patients had higher vagal modulation than the survived ARDS patients. Further, Firth logistic regression analysis identified HFP and TP as the independent predictors of mortality in ARDS patients on admission to the SICU (Table 4). The HFP, an indicator of vagal modulation, appeared to be the best predictor of mortality in ARDS patients (*p* = 0.02). Thus the HFP might be useful in the monitoring and prediction of outcome in various critically ill patients, including ARDS, and be used in the prediction of mortality on their admission to the SICU.

The mechanism underlying the increase in vagal modulation in critically ill patients has not been well understood yet. It has been shown that direct electrical stimulation of the peripheral vagus nerve in vivo during lethal endotoxaemia in rats could inhibit tumor necrosis factor (TNF) synthesis in liver, attenuated peak serum TNF amounts, and prevented the development of shock [37]. Thus, the increased vagal modulation in ARDS patients might be one of the pathophysiological responses of the patient to counteract the cytokines synthesis, attenuate the amount of cytokines in the serum, and prevent the cytokines-induced systemic inflammation in ARDS. Since the non-survived ARDS patients had increased vagal modulation relative to the survived ARDS patients, the use of anticholinergic and sympathomimetic agents to correct their autonomic dysfunction might be considered in patients with ARDS after thoracic surgery. Further studies are warranted to investigate the underlying mechanism of increased vagal modulation in critically ill patients, including ARDS.

The HRV parameters in ARDS patients can be influenced by many interventions performed in the ICU, such as sedation, catecholamine therapy, volume management, ventilator settings, etc. Previous study has indicated that the increase in V_T_ can increase the HFP component of HRV [26]. To minimize the effect of V_T_ on the indicator of vagal modulation, a new indicator nHFP/V_T_ was used to represent the V_T_-corrected indicator of vagal modulation. It was found that the ARDS patients still had higher vagal modulation than the non-ARDS patients. Some studies had reported that midazolam and inotropes might change the autonomic nervous activities of the patients [38, 39]. Though the midazolam and norepinephrine were used more frequently in the ARDS patients than the non-ARDS patients, the use of midazolam and norepinephrine were not significantly different between survivors and non-survivors of ARDS patients. Thus, the significantly increase in vagal modulation in the non-survivors of ARDS should not be caused by the use of midazolam and norepinephrine. In ARDS patients, PEEP has been widely used to improve arterial oxygenation; however, the increase in PEEP can reduce the baroreflex sensitivity, and can alter the autonomic nervous function in these patients [40]. The present study found that ARDS patients required higher PEEP during mechanical ventilation than the non-ARDS patients. Since the level of PEEP was not significantly different between the survivors and non-survivors of ARDS patients, the significantly increased vagal modulation in the non-survivors of ARDS patients should not be caused by the use of higher PEEP in those patients.

The major limitation of this study was the small number of ARDS patients. Since the incidence of ARDS is not very high in the post-operative patients with lung or esophageal cancer, it was not easy to collect a large number of cases for statistical analysis in a study period of 2 years.

## Conclusions

The vagal modulation of thoracic surgical patients with ARDS was enhanced compared with non-ARDS patients. Non-survived ARDS patients had higher vagal activity than survived ARDS patients. The vagal modulation-related parameter such as HFP and TP might be used as predictors to identify ARDS patients with high risk of mortality on admission to the SICU, especially the HFP. Increased vagal modulation might be an indicator for poor prognosis in critically ill patients following thoracic surgery, and anticholinergic and sympathomimetic agents might be tried to correct the autonomic dysfunction of ARDS patients.

## Abbreviations

AaDO_2_: Alveolar-arterial oxygen difference
ALIS: Acute lung injury score
APACHE: Acute Physiology and Chronic Health Evaluation
ARDS: Acute respiratory distress syndrome
ASA: American Society of Anesthesiologists
Cdyn: Dynamic compliance
COPD: Chronic obstructive pulmonary disease
CV_RR_: Coefficient of variation of RRI
ECG: Electrocardiographic
ECMO: Extracorporeal membrane oxygenation
EPV − 10: 10 events per variable
FIO_2_: Fraction of inspired oxygen
HCO_3_^−^: Bicarbonate
HFP: High-frequency power
HRV: Heart rate variability
I/O: Intake/output
ICU: Intensive care unit
LF: Low-frequency
LFP: Low-frequency power
LHR: Low-/high-frequency power ratio
mRRI: Mean RR interval
MV: Minute ventilation
nHFP: Normalized HFP
nLFP: Normalized LFP
nVLFP: Normalized VLFP
PaCO_2_: Arterial partial pressure of carbon dioxide
PaO_2_: Arterial partial pressure of oxygen
PEEP: Positive end-expiratory pressure
P_insp_: Inspiratory pressure
PSD: Power spectral density
RASS: Richmond Agitation-Sedation Scale
RMSSD: Root mean squared successive difference of RRI
ROC: Receiver operating characteristic
RR: Respiratory rate
RRI: RR intervals
SDNN: Standard deviation of normal-to-normal QRS intervals
SD_RR_: Standard deviation of RRI
SICU: Surgical intensive care unit
T_insp_: Inspiration time
TNF: Tumor necrosis factor
TP: Total power
VATS: Video-assisted thoracic surgery
VLFP: Very low-frequency power
V_T_: Tidal volume
